# Interpreting Xpert MTB/RIF Ultra in a High-Risk Tuberculosis Referral Cohort in Taiwan: Treatment-Associated Culture Discordance and Rifampicin-Indeterminate Results

**DOI:** 10.3390/diagnostics16213651

**Published:** 2026-11-04

**Authors:** Ming-Jr Jian, Hsing-Yi Chung, Tai-Han Lin, Chih-Kai Chang, Cherng-Lih Perng, Hung-Sheng Shang

**Affiliations:** 1 Division of Clinical Pathology, Department of Pathology, Tri-Service General Hospital, National Defense Medical University, Taipei 11490, Taiwan; mj0106@gmail.com (M.-J.J.); cindyft12@gmail.com (H.-Y.C.); garlicbun84@gmail.com (T.-H.L.); mblkaiser@gmail.com (C.-K.C.); 2 Graduate Institute of Pathology and Parasitology, National Defense Medical University, Taipei 11490, Taiwan

**Keywords:** Xpert MTB/RIF Ultra, *Mycobacterium tuberculosis* complex, rifampicin resistance, disputed *rpoB* mutations, line-probe assay, paucibacillary tuberculosis, diagnostic stewardship

## Abstract

**Background/Objectives:** Interpretation of positive and indeterminate rifampicin (RIF) calls from Xpert MTB/RIF Ultra (Ultra) in previously treated, high-risk populations is poorly characterised. **Methods:** We retrospectively analysed all 2271 Ultra records (2024) at Taiwan’s National Reference Mycobacteriology Laboratory, a high-risk referral cohort, against referring-laboratory culture and phenotypic drug-susceptibility testing (pDST), with line-probe assay (LPA) as a reflex test and treatment history defined by referral indication. **Results:** Among 1662 culture-paired records, sensitivity was 91.7% and specificity 75.1%. Specificity was 92.7% in screening or risk-based referrals but 56.4% in previously or currently treated patients, who accounted for 84.8% of culture-negative Ultra-positive results. Positive predictive value fell from 89.3% (HIGH) to 36.2% (Trace). All 77 Trace results were RIF indeterminate (77 of 78 indeterminate calls). In 362 of 382 pDST-paired records with determinate calls, RIF sensitivity and specificity were 94.3% and 96.8%. Eight of 10 pDST-discordant resistance calls, none sequenced, were LPA-resistant. Reflex LPA was interpretable in 98.9% of resistance-detected records but in 19.2% of indeterminate records. **Conclusions:** Low specificity was concentrated among previously or currently treated patients, and indeterminate calls reflected Trace detection. LPA supported most pDST-discordant resistance calls, whose mutational basis remains unconfirmed. Stewardship should reflect the limited yield of reflex LPA in paucibacillary specimens.

## 1. Introduction

Tuberculosis (TB) remains among the leading infectious causes of death worldwide, and rapid detection of *Mycobacterium tuberculosis* complex (MTBC) together with rifampicin (RIF) resistance underpins both treatment decisions and infection control [1]. Cartridge-based nucleic acid amplification has become the reference front-line approach. The first-generation Xpert MTB/RIF assay reported MTB and RIF results within approximately two hours, but its analytical limit of detection of about 131 CFU/mL limited sensitivity in smear-negative and extrapulmonary disease [2,3].

Xpert MTB/RIF Ultra (Ultra) lowered the limit of detection to approximately 16 CFU/mL by targeting the multicopy insertion sequences IS6110 and IS1081 and by adding melting-temperature-based *rpoB* analysis [3,4]. In a Cochrane review of direct comparisons in adults, Ultra was 6.3% more sensitive and 2.7% less specific than Xpert MTB/RIF, with a higher proportion of RIF-indeterminate results [5]. A parallel review in children found Ultra accurate for detecting pulmonary TB in sputum, gastric aspirate and stool and noted that Trace results were common, but the data on rifampicin resistance were too sparse for firm conclusions [6]. Service evaluations in low-incidence European settings found that Ultra raised sensitivity for smear-negative pulmonary TB substantially, with a modest reduction in specificity [7,8], and a prospective multicentre study confirmed comparable behaviour under high-burden conditions [9].

Two features of the Ultra design shape how its RIF result should be read, and both are frequently underappreciated in clinical practice. First, MTBC detection relies on multicopy targets whereas RIF resistance is inferred from the single-copy *rpoB* gene, so the bacillary load required for a reliable resistance call is higher than that required for detection. The manufacturer’s instructions for use accordingly specify that every MTB Trace result is reported as RIF Indeterminate. Second, melting-temperature signatures detect any deviation within the *rpoB* resistance-determining region, including mutations such as L511P, D516Y, H526N and L533P that confer only low-level resistance and are frequently classified as susceptible by phenotypic drug-susceptibility testing (pDST) at standard critical concentrations, particularly in automated liquid culture [10,11]. These so-called disputed mutations are clinically relevant, are associated with poor outcomes on standard regimens, and are catalogued by the World Health Organization as resistance-associated [12]. Because phenotypic methods detect these mutations poorly, sequencing is the appropriate arbiter when molecular and phenotypic results disagree, and WHO has endorsed targeted next-generation sequencing (tNGS) for the detection of drug-resistant TB [13]. Its value in paucibacillary specimens is, however, constrained by the same limited input DNA that affects other molecular assays.

Most published Ultra evaluations report aggregate sensitivity and specificity for mixed presumptive-TB populations. Far less is known about how Ultra behaves in a referral population enriched for previously treated patients, in which both culture and pDST are imperfect comparators, and about whether the reflex testing routinely recommended for positive or indeterminate RIF calls is actually achievable in the specimens that generate those calls. We therefore analysed the complete 2024 Ultra dataset of Taiwan’s National Reference Mycobacteriology Laboratory, with three objectives: to quantify how referral-defined treatment history and bacillary load relate to Ultra specificity and positive predictive value; to characterise discordant and indeterminate RIF calls using reflex line-probe assay (LPA) and pDST; and to propose, for future evaluation, a diagnostic-stewardship approach that reflects the observed feasibility of reflex testing.

## 2. Materials and Methods

### 2.1. Study Design and Setting

This retrospective diagnostic-accuracy study used specimen-record-level data from all Ultra tests performed between 1 January and 31 December 2024 at the National Reference Mycobacteriology Laboratory, Tri-Service General Hospital, Taipei, Taiwan, linked to the smear, culture and phenotypic drug-susceptibility results reported by the referring laboratories to the national tuberculosis information system of the Taiwan Centers for Disease Control. The laboratory receives respiratory specimens referred nationwide from hospitals and public health centres for patients who meet at least one nationally defined high-risk criterion. These criteria are: treatment failure, defined as persistent smear or culture positivity after five or more months of first-line chemotherapy; culture positivity two or more months after treatment initiation; relapse after documented cure or treatment completion; loss to follow-up during a prior TB episode; close contact with a culture-positive multidrug-resistant TB (MDR-TB) index patient; rapid molecular evidence of RIF resistance obtained at a referring hospital; origin from or residence in a WHO-defined MDR-TB high-burden country with at least six months of residence before presentation; residence in a domestic high-incidence district defined by the Taiwan Centers for Disease Control; application for second-line (Group A or B) anti-TB drugs; recommendation by a clinical TB review committee; occupational exposure to zoonotic *Mycobacterium* species among livestock or abattoir workers; suspected or confirmed *Mycobacterium bovis* infection; or latent TB infection progressing to active disease.

Because the objective was to characterise real-world laboratory performance across the complete annual dataset, duplicate submissions were retained at the specimen-record level unless the relevant Ultra, culture or pDST field was missing or non-interpretable for a given analysis. The 2271 records originated from 2021 unique patients. A patient-level sensitivity analysis restricted to the first eligible specimen per patient within each analysis population, ordered by date of receipt and laboratory accession number, is provided in Supplementary Tables S1–S3.

The data were generated by the National Reference Mycobacteriology Laboratory, which performs statutory tuberculosis reference testing under commission from the Taiwan Centers for Disease Control. Ethics committee review was therefore not required under Announcement No. 1010265075 (5 July 2012) of the Department of Health (now the Ministry of Health and Welfare), Taiwan, issued under the Human Subjects Research Act, which exempts from ethics committee review public policy evaluation research conducted by, or on behalf of, a public agency performing its statutory duties, and the requirement for informed consent was waived under Announcement No. 1010265083 (5 July 2012) of the same authority. All records were rendered innominate before extraction, and the study was conducted in accordance with the Declaration of Helsinki.

### 2.2. Specimen Processing and Xpert MTB/RIF Ultra

Respiratory specimens were decontaminated and concentrated by the referring laboratories, and the resulting sediment was sent to the study laboratory, where it was used for Ultra testing. Acid-fast smear grades (negative, scanty, 1+, 2+, 3+ or 4+) were those reported by the referring laboratories according to national guidelines.

Sediment was processed with Xpert MTB/RIF Ultra cartridges on the GeneXpert platform according to the manufacturer’s instructions. MTBC results were reported as MTB Detected with a semiquantitative category (HIGH, MEDIUM, LOW or VERY LOW), MTB Trace Detected, or MTB Not Detected. MTB Trace Detected was not assigned a semiquantitative category. RIF results were reported as RIF Resistance Detected, RIF Resistance Not Detected, or RIF Resistance Indeterminate, together with the *rpoB* probe and melting-peak annotation generated by the instrument.

### 2.3. Mycobacterial Culture

Mycobacterial culture was not performed by the study laboratory. Culture was performed by the referring hospitals as part of routine diagnostic care, either on the same specimen or on another specimen from the same patient, and the results entered by those laboratories into the national tuberculosis information system were extracted for this study. Each Ultra record was linked to the culture result entered in the system under the same referral record. The system also recorded whether that culture had been performed on the same specimen as the Ultra test or on another specimen from the same patient; this information was not used to restrict the accuracy analysis. Culture results were recorded as MTBC, non-tuberculous mycobacteria (NTM), negative, contaminated, or not performed; records for which no culture result could be retrieved were classified as having no culture record or as untraceable.

### 2.4. Phenotypic Drug-Susceptibility Testing

Phenotypic drug-susceptibility testing (pDST) was likewise performed by the referring laboratories, and the rifampicin result entered by each laboratory into the national tuberculosis information system was used. The pDST method and critical concentrations were determined by each performing laboratory and were not recorded in the national system. Because pDST requires a culture isolate, a pDST result could be available only for records with culture growth.

### 2.5. Reflex Line-Probe Assays

Specimens with RIF Resistance Detected or RIF Resistance Indeterminate were routed for reflex testing with GenoType MTBDRplus v2.0 and GenoType MTBDRsl v2.0 (Hain Lifescience, Nehren, Germany). LPA was also performed outside the reflex rule on 54 RIF Resistance Not Detected records, predominantly those referred for second-line drug application (*n* = 48). Line blots were read independently by two trained technologists; discordant reads were referred to a third senior reader whose interpretation was final. LPA RIF results were recorded as resistant, susceptible, or equivocal when band intensity did not meet interpretive criteria; equivocal results were not considered interpretable. Reflex testing was not always completed, principally because insufficient residual sediment remained or because amplification failed in paucibacillary specimens; the proportion of records with an interpretable reflex result was reported as an outcome of the workflow, encompassing both specimens that could not be tested and failed assays, rather than as an analytical failure rate of LPA.

### 2.6. Sequencing

Sequencing was not part of the routine diagnostic workflow. Where it had been performed to resolve a molecular rifampicin result, the codon-level finding and its interpretation were recorded in the laboratory information system and were extracted for this study. These records are reported descriptively and were not used as a reference standard.

### 2.7. Reference Standards and Analysis Populations

Mycobacterial culture, as reported by the referring laboratories, was the reference standard for MTBC detection, and pDST was the reference standard for RIF resistance. Accuracy estimates are therefore relative to culture and pDST results as routinely reported to the national system, rather than to a standardised reference applied to the specimen tested by Ultra. Analytic populations were defined explicitly because reference-standard availability differed by outcome (Figure 1). The descriptive population comprised records with an interpretable Ultra MTBC result (*n* = 2156). The MTBC accuracy population comprised records with both an interpretable Ultra result and a valid culture comparator (*n* = 1662); culture results recorded as contaminated, not performed, or untraceable, and records with no culture record, were excluded, and NTM growth was classified as MTBC-negative. The RIF accuracy population comprised MTBC-positive records with a paired pDST rifampicin result (*n* = 382); the primary RIF analysis excluded the 20 records with an indeterminate call (*n* = 362). The availability of culture and pDST comparators is described in Section 3.1.

For the stratified analysis of treatment history, referral categories denoting current or previous anti-TB treatment (treatment failure, culture positivity at two months or more, relapse or re-treatment, application for second-line drugs, RIF resistance detected before referral, and loss to follow-up) were grouped as previously or currently treated; the remaining categories, which denote epidemiological or occupational risk without a documented prior treatment episode, were grouped as screening or risk-based referral. Recommendations by a clinical TB review committee were assigned to this group because the referral indication did not document a treatment episode. This grouping is a pragmatic proxy based on the referral indication recorded at the time of testing rather than on individual treatment records, and the screening or risk-based group should not be read as verified treatment-naive. Two categories in the treated group, application for second-line drugs (*n* = 158) and RIF resistance detected before referral (*n* = 85), do not by themselves establish a treatment episode.

### 2.8. Statistical Analysis

Sensitivity, specificity, positive predictive value (PPV), negative predictive value (NPV) and overall accuracy were calculated with Wilson score 95% confidence intervals (CIs). RIF Resistance Indeterminate results were excluded from the primary RIF accuracy analysis and, in a scenario analysis, treated as non-detected; this scenario is an analytical assumption and does not imply that an indeterminate call can be interpreted as susceptible. Proportions were compared with the chi-squared test, with two-sided *p* < 0.05 considered significant. Comparisons between treatment-history groups and between semiquantitative categories were unadjusted. Statistical analyses were performed using IBM SPSS Statistics for Windows, version 22.0 (IBM Corp., Armonk, NY, USA).

## 3. Results

### 3.1. Cohort Composition

A total of 2271 Ultra specimen records were processed in 2024. Referral categories are shown in Table 1. Epidemiological risk accounted for the largest share, with 587 records (25.8%) from patients originating from or residing in high MDR-TB-burden countries and 555 (24.4%) from domestic high-incidence districts. Categories denoting current or previous treatment together accounted for 981 records (43.2%).

Ultra returned an interpretable MTBC result in 2156 records (94.9%); 115 records (5.1%) were missing or non-interpretable. Among interpretable results, 856 (39.7%) were MTBC-positive, comprising 779 MTB Detected and 77 MTB Trace Detected. The flow of records through each analysis is shown in Figure 1.

A valid culture comparator was available for 1662 of the 2156 records with an interpretable Ultra result (77.1%); the remaining 494 had no culture record (*n* = 443), were untraceable (*n* = 27), were not cultured (*n* = 17) or were contaminated (*n* = 7) (Figure 1). Availability was higher for Ultra-positive than for Ultra-negative records (695 of 856, 81.2%, vs. 967 of 1300, 74.4%; *p* < 0.001), and among Ultra-positive records it ranged from 85.7% for HIGH to 75.3% for Trace results (*p* = 0.30). Among all 2271 records, the proportion included in the culture-paired analysis, which also required an interpretable Ultra result, was 78.2% (767 of 981) for previously or currently treated patients and 69.4% (895 of 1290) for screening or risk-based referrals (*p* < 0.001). A paired pDST result was available for 382 of the 386 culture-positive Ultra-positive records (99.0%). The 474 MTBC-positive records without pDST comprised 309 records whose culture was negative for MTBC (including NTM growth), so that no MTBC isolate was available for pDST, 161 without a culture comparator and 4 for which pDST was not issued. pDST availability therefore followed MTBC culture growth and differed by Ultra RIF call: 60 of 87 RIF Resistance Detected (69.0%), 302 of 691 RIF Resistance Not Detected (43.7%) and 20 of 78 RIF Resistance Indeterminate records (25.6%; *p* < 0.001).

### 3.2. MTBC Detection Accuracy

In the culture-paired population (*n* = 1662), Ultra yielded 386 true-positive, 309 false-positive, 35 false-negative and 932 true-negative results, corresponding to 91.7% sensitivity (95% CI 88.7–94.0), 75.1% specificity (95% CI 72.6–77.4), 55.5% PPV (95% CI 51.8–59.2), 96.4% NPV (95% CI 95.0–97.4) and 79.3% overall accuracy (95% CI 77.3–81.2). Sensitivity rose with smear grade, from 73.1% in smear-negative specimens and 91.2% in scanty specimens to 97.8% to 100.0% in 1+ to 4+ specimens (Table 2). Results were stable in the patient-level sensitivity analysis restricted to the first eligible specimen per patient (*n* = 1490): sensitivity 91.0% (95% CI 87.8–93.5), specificity 77.2% (95% CI 74.6–79.6), PPV 58.6% (95% CI 54.6–62.4) and NPV 96.0% (95% CI 94.5–97.1) (Supplementary Table S1). At the patient level, specificity was 92.8% (95% CI 90.4–94.6) in screening or risk-based referrals and 59.6% (95% CI 55.3–63.7) in previously or currently treated patients (Supplementary Table S2). Of 355 patients with a paired pDST result, 335 had a determinate RIF call, and RIF sensitivity and specificity were 95.3% (95% CI 84.5–98.7) and 97.3% (95% CI 94.7–98.6) (Supplementary Table S3).

### 3.3. Specificity and Positive Predictive Value Differ by Referral-Defined Treatment History

Specificity and PPV differed substantially between treatment-history groups (Table 3). Among screening or risk-based referrals, specificity was 92.7% (95% CI 90.4–94.4) and PPV was 83.0% (95% CI 78.1–86.9). Among previously or currently treated patients, specificity fell to 56.4% (95% CI 52.4–60.3) and PPV to 37.5% (95% CI 33.0–42.2) (*p* < 0.001 for both comparisons). Of the 309 culture-negative Ultra-positive results in the full culture-paired population, 262 (84.8%) arose in the previously or currently treated group, with the largest contributions from culture positivity at two months or more (*n* = 115) and treatment failure (*n* = 70), two categories that directly document a treatment episode. Sensitivity and NPV were comparable between groups. These comparisons are unadjusted for bacillary load and smear grade.

### 3.4. Positive Predictive Value by Semiquantitative Bacillary Load

Among the 695 Ultra-positive records in the culture-paired population, PPV declined monotonically with decreasing semiquantitative load, from 89.3% for HIGH results to 36.2% for Trace results (Table 4). LOW, VERY LOW and Trace results together accounted for 506 of 695 Ultra-positive records (72.8%) and for 272 of the 309 culture-negative Ultra-positive results (88.0%).

### 3.5. Rifampicin Calls by Bacillary Load

Across all 856 MTBC-positive records, Ultra reported RIF Resistance Detected in 87 (10.2%), RIF Resistance Not Detected in 691 (80.7%) and RIF Resistance Indeterminate in 78 (9.1%). The distribution of RIF calls by semiquantitative category was strictly load-dependent (Table 5). Every one of the 77 MTB Trace results was reported as RIF Resistance Indeterminate, with no Trace record receiving a Detected or Not Detected call. Only one indeterminate result occurred outside the Trace category, in a VERY LOW specimen. Conversely, no HIGH, MEDIUM or LOW record was reported as indeterminate.

### 3.6. Rifampicin Accuracy Against Phenotypic DST

A paired pDST rifampicin result was available for 382 MTBC-positive records (Figure 1). RIF Resistance Detected was confirmed by pDST in 50 of 60 results (83.3%). RIF Resistance Not Detected was recorded in 302 results, of which 299 were pDST-susceptible and 3 pDST-resistant. Twenty results were RIF Resistance Indeterminate, all of them Trace specimens, and only one of these was pDST-resistant.

After exclusion of the 20 indeterminate results (*n* = 362), Ultra detected RIF resistance with 94.3% sensitivity (95% CI 84.6–98.1), 96.8% specificity (95% CI 94.1–98.2), 83.3% PPV (95% CI 72.0–90.7), 99.0% NPV (95% CI 97.1–99.7) and 96.4% accuracy (95% CI 94.0–97.9). In a scenario analysis in which indeterminate results were treated as non-detected (*n* = 382), sensitivity was 92.6% (95% CI 82.4–97.1), specificity 97.0% (95% CI 94.5–98.3), PPV 83.3% (95% CI 72.0–90.7) and NPV 98.8% (95% CI 96.8–99.5).

Notably, the 10 pDST-discordant RIF Resistance Detected results were not concentrated in paucibacillary specimens. They occurred in HIGH (*n* = 3), MEDIUM (*n* = 1), LOW (*n* = 3) and VERY LOW (*n* = 3) specimens, and 7 of the 10 were smear-positive. The three false-negative results likewise occurred in HIGH (*n* = 2) and LOW (*n* = 1) specimens; one of the HIGH records was also susceptible by LPA, and the other two had no LPA result.

### 3.7. Molecular Findings in Rifampicin-Discordant Records

None of the 10 pDST-discordant RIF Resistance Detected records was sequenced, so the mutations underlying these discordances, and the reasons for discordance, could not be determined; the findings in this section are descriptive. A reflex LPA result was available for all 10 records and an Ultra *rpoB* melting annotation for nine (Table 6). Eight of the 10 were also classified as RIF-resistant by LPA, with probe patterns comprising loss or weakening of *rpoB* wild-type bands (WT1, WT3 or WT8) or gain of MUT2A. The melting annotations were *rpoB*1 in six records and *rpoB*4 in three records.

Considering all MTBC-positive records with both an LPA and a pDST rifampicin result (*n* = 85, including 21 RIF Resistance Not Detected records tested outside the reflex rule), the two molecular methods agreed with each other far more closely than either agreed with pDST: LPA classified 46 of 51 pDST-resistant isolates as resistant, but also classified 10 of 34 pDST-susceptible isolates as resistant, giving an LPA positive predictive value against pDST of 82.1%, closely matching the 83.3% PPV of Ultra. Because Ultra and LPA interrogate the same resistance-determining region, this concordance is compatible with the presence of *rpoB* mutations but does not by itself adjudicate between genotype and phenotype, nor does it exclude differences between the specimens tested by Ultra and by the referring laboratories.

Further context comes from seven other records in the same cohort in which sequencing was performed outside the routine workflow, and the testing laboratory reported a disputed-mutation classification (Table 7); none of these is among the 10 discordant records. Six of these carried a documented codon change: L511P in two records, L533P in two records, D516A in one and S531F in one (*Escherichia coli* numbering; equivalent to L430P, L452P, D435A and S450F in *M. tuberculosis* numbering). Of these, only L511P and L533P correspond to the WHO borderline mutations L430P and L452P, whereas D516A and S531F are not among the mutations listed as borderline [12]. In the seventh record, no codon change was specified; the phenotypic rifampicin result was superseded by a tNGS-adjudicated disputed-mutation classification, and this record was therefore not eligible for the paired pDST analysis. All seven were RIF Resistance Detected by Ultra and resistant by LPA, and four also carried the *katG* MUT1 marker of isoniazid resistance.

In the six records with a documented codon, the melting-peak annotation corresponded to the sequenced codon region: both *rpoB*1 records carried L511P, both *rpoB*4 Mut melt B records carried L533P, and the *rpoB*2 and *rpoB*3 records carried D516A and S531F, respectively. Nine of the 10 pDST-discordant records carried an *rpoB*1 or *rpoB*4 annotation, but their LPA patterns matched those of the sequenced records only for *rpoB*4. The three *rpoB*4 Mut melt B discordant records shared the complete profile of the two L533P records, comprising loss or weakening of WT8 and the *katG* MUT1 marker; this similarity is compatible with L533P but does not identify the mutation. In contrast, none of the six *rpoB*1 discordant records showed the WT2 weakening seen with sequenced L511P: three showed loss or weakening of WT1 (codons 505 to 509), two showed no band loss, and one showed MUT2A, a band corresponding to H526Y that is not expected with an *rpoB*1 annotation. The mutations underlying the *rpoB*1 discordances therefore remain unresolved. Of the six records with a documented codon, three had a paired pDST result and all three were phenotypically resistant, so the cohort provides no direct example of a sequence-confirmed mutation classified as susceptible by pDST; the remaining three were culture-negative and therefore had no phenotypic comparator.

### 3.8. Feasibility of Reflex Line-Probe Testing

Of the 87 records with RIF Resistance Detected, 86 (98.9%) yielded an interpretable reflex LPA rifampicin result (77 resistant, 9 susceptible). In contrast, of the 78 records with RIF Resistance Indeterminate, an LPA result was obtained in only 18 (23.1%), comprising 3 resistant, 12 susceptible and 3 equivocal, so that an interpretable result was available in 15 (19.2%); the remaining 60 (76.9%) yielded no LPA result (Table 8), principally because of insufficient residual sediment or amplification failure. All three equivocal LPA results occurred in Trace specimens. LPA performed outside the reflex rule on 54 RIF Resistance Not Detected records was susceptible in all 54.

## 4. Discussion

In this complete-year reference-laboratory dataset, Ultra performed as a highly sensitive front-line test for MTBC with a high NPV for rifampicin resistance among records with paired pDST. The more informative findings, however, concern how its positive and indeterminate results should be interpreted in a population enriched for previously treated patients.

The overall specificity of 75.1% is considerably lower than that reported in most Ultra evaluations, and the stratified analysis suggests why. Specificity was 92.7% in screening and epidemiological-risk referrals, which is consistent with published pooled estimates [14], but 56.4% in patients with a current or previous treatment episode, who contributed 85% of all culture-negative Ultra-positive results. The two referral categories that contributed most, culture positivity at two months or more and treatment failure, by definition comprise patients receiving anti-TB therapy at the time of sampling. Detection of residual or non-viable *M. tuberculosis* DNA in such patients is a well-recognised phenomenon [15] and a plausible contributor, but it was not confirmed in individual specimens. Some discordances may instead reflect the reference standard, because culture was performed by the referring laboratories, not necessarily on the specimen tested by Ultra, and growth may fail in patients receiving treatment. The association is unadjusted and observational: because treatment history and bacillary load were not modelled jointly, their separate contributions cannot be distinguished, and the data do not show that treatment caused the discordance. The monotonic decline of PPV with semiquantitative load, from 89.3% at HIGH to 36.2% at Trace, is consistent with the same pattern, and a 2025 Cochrane update similarly found that only 38.8% of Trace-positive results were true positives [14]. PPV depends on the prevalence of culture-positive disease and on the composition of the tested population, and here it was measured against culture positivity rather than active disease. The PPVs reported here, including that for Trace results, apply to this referral cohort and should not be compared directly with those from populations with different referral criteria or prevalence. For the same reason, aggregate specificity figures from enriched referral populations should not be compared directly with those from presumptive-TB cohorts, and laboratories serving referral networks should report Ultra positivity with the semiquantitative category and the treatment context attached.

The distribution of RIF Indeterminate results was entirely determined by bacillary load. All 77 Trace results were reported as indeterminate, and only one indeterminate call arose outside the Trace category. This is not an incidental observation but a direct consequence of assay design: Ultra detects MTBC using the multicopy IS6110 and IS1081 targets while inferring resistance from single-copy *rpoB*, and the manufacturer specifies that Trace results are reported as RIF Indeterminate. Framing indeterminate calls as an unexplained performance problem is therefore misleading. The clinically relevant questions are how large the resulting reflex workload is and whether that workload can be discharged. In our dataset, indeterminate calls represented 9.1% of MTBC-positive records, and only one of the 20 with paired pDST proved rifampicin-resistant, a result consistent with nationwide data from Rwanda showing that most Ultra rifampicin-resistance calls in paucibacillary specimens are not confirmed by repeat testing, sequencing or pDST [16]. An indeterminate call nevertheless does not exclude resistance, and because only 20 of the 78 indeterminate records had a paired pDST result, the proportion of resistance among them is imprecisely estimated.

The 10 RIF Resistance Detected results classified as susceptible by pDST require cautious interpretation. These were not concentrated in low-burden specimens, and 8 of the 10 were also classified as resistant by LPA. Mutations such as L511P, D516Y, H526N, H526L and L533P confer low-level resistance that is readily missed by growth-based methods, most markedly in automated liquid culture but also in solid-medium proportion testing: in a large retreatment cohort, approximately one in five strains carrying a disputed mutation tested susceptible on Löwenstein–Jensen proportion DST, with L511P among the mutations most often missed [10,11]. Patients harbouring these mutations have poor outcomes when treated with standard rifampicin-containing regimens, and WHO classifies them as resistance-associated [12]. Disputed mutations are therefore one possible explanation for the discordance, but our data cannot confirm it. None of the 10 discordant records was sequenced; Ultra and LPA interrogate the same *rpoB* region, so their agreement is not independent confirmation; and the similarity between the three *rpoB*4 discordant records and the sequenced L533P records is a descriptive observation that requires sequencing to confirm. Within the cohort, all three sequenced records with a paired pDST result were phenotypically resistant, so the evidence that growth-based testing misses such mutations derives from external cohorts [10,11]. Differences between the specimens tested by Ultra and by the referring laboratories, and variation in pDST methods and critical concentrations, which were not recorded, may also contribute. We therefore do not estimate the extent to which the 83.3% PPV of Ultra for rifampicin resistance reflects pDST limitations. Conversely, two of the three pDST-resistant results missed by Ultra occurred in HIGH-burden specimens where a detection failure is unlikely, and one of them was also susceptible by LPA, raising the possibility of resistance conferred by mutations outside the *rpoB* resistance-determining region, such as I491F, which commercial molecular assays do not detect [17].

Our feasibility data qualify the reflex-testing recommendations that commonly accompany Ultra implementation. Reflex LPA produced an interpretable rifampicin result in 98.9% of RIF Resistance Detected records but in only 19.2% (15 of 78) of RIF Resistance Indeterminate records; an LPA result of any kind, including 3 equivocal, was obtained in 18 (23.1%). In this workflow, therefore, most indeterminate records did not yield an interpretable reflex LPA result, and a recommendation to reflex all indeterminate results to LPA on the same residual specimen is difficult to achieve in practice. Because records with no LPA result include specimens that could not be tested, owing to insufficient residual sediment as well as failed assays, this yield describes the workflow rather than the analytical performance of LPA, and it does not show that testing a fresh specimen would be unproductive. Phenotypic confirmation, which depends on culture growth, and targeted next-generation sequencing, which depends on input DNA, may face similar constraints in paucibacillary specimens, but neither was systematically evaluated here. WHO has endorsed targeted sequencing for drug-resistant TB detection [13], and it is the appropriate arbiter for discordant results in adequately loaded specimens; its yield in Trace-level material remains to be established. Our proposed algorithm reflects this by routing Trace-derived indeterminate results to clinical assessment, culture and, when clinically indicated, a fresh specimen rather than to an additional molecular test on the same specimen.

Latent TB infection progressing to active disease accounted for 79 referrals. Patients in this category typically have low bacillary burdens, and their inclusion would be expected to reduce sensitivity while contributing Trace-level and low-load positives with a higher probability of being culture-negative. This is one component of the enriched, heterogeneous composition that distinguishes a reference-laboratory cohort from a presumptive-TB cohort.

On the basis of these findings, we propose the diagnostic-stewardship approach shown in Figure 2. It was derived retrospectively from a single national referral laboratory, has not been prospectively validated, and its effects on treatment decisions, patient outcomes, cost and safety were not evaluated; it is intended as a basis for further evaluation rather than as an established management pathway. MTBC-positive results with RIF Resistance Not Detected had an NPV of 99.0% against pDST, but this estimate derives from the 302 such records with a paired pDST result; management should follow national guidance, with culture and pDST completed as usual, particularly when the risk of resistance is high. RIF Resistance Detected results should be routed to reflex LPA and pDST, and a pDST-susceptible result that is contradicted by a concordant molecular resistance call should not be dismissed as a false positive but considered for sequencing, where available, as a possible disputed mutation. RIF Resistance Indeterminate results arising from Trace detection do not exclude resistance. Because reflex LPA did not yield an interpretable result in more than three-quarters of such records, routine reflex LPA testing of the same low-load specimen is unlikely to resolve them; management should instead be guided by clinical severity, transmission risk, and the culture result, with a fresh specimen for repeat rapid molecular testing considered when clinically indicated or when the risk of resistance is high, and pDST performed once growth is available. Ultra-positive but culture-negative results in previously treated patients should be interpreted with caution, because they may reflect residual or non-viable nucleic acid or differences between specimens rather than active bacteriological disease, particularly when the semiquantitative category is LOW, VERY LOW or Trace, and should be correlated with clinical findings.

This study has several limitations. First, the analysis was performed at the specimen-record level and retained duplicate submissions, which may over-represent patients undergoing repeated testing; the patient-level sensitivity analysis gave similar estimates (Supplementary Tables S1–S3). Second, the treatment-history grouping relies on the referral indication documented at the time of testing rather than on verified individual treatment records, so some misclassification is likely. Its direction cannot be determined from these data. If treatment is associated with culture-negative Ultra positivity, untreated patients classified in the treated group would tend to raise specificity in that group, and treated patients classified as screening referrals would tend to lower specificity in the screening group; both would narrow the observed contrast, but the net effect depends on the results of the misclassified records, which are unknown. The two treated categories that do not by themselves document a treatment episode, application for second-line drugs and RIF resistance detected before referral (243 of 981 treated records), are the most likely sources of such misclassification. We did not repeat the analysis after excluding these two categories, so whether the specificity contrast persists without them was not verified. The group comparisons were also unadjusted, so the contribution of treatment history independent of bacillary load and smear grade could not be determined. Third, specimen type was not captured as a discrete field in the laboratory dataset. Respiratory specimens comprised expectorated sputum and airway lavage sediment, and Ultra is labelled for sputum, so results for lavage specimens should be regarded as reflecting off-label use; performance could not be compared between specimen types. Fourth, the reference standards were not standardised. Culture and pDST were performed by the referring laboratories using methods and critical concentrations that were not recorded, not necessarily on the specimen tested by Ultra, and their results were entered by those laboratories into the national system. Some Ultra-positive, culture-negative results may therefore reflect differences between specimens or culture failure rather than residual DNA, and some rifampicin discordances may reflect specimen or method differences rather than assay error or disputed mutations. Fifth, reference results were incomplete. A culture comparator was unavailable for 494 records with an interpretable Ultra result, mostly because no culture had been recorded, and pDST was available only for records with MTBC culture growth and therefore more often for RIF Resistance Detected than for indeterminate calls. If records without a reference result differ in disease or resistance status, the estimates may be affected by verification bias, and the RIF accuracy estimates apply to records with MTBC culture growth. Sixth, sequencing was performed only sporadically and outside the routine workflow, and none of the 10 pDST-discordant records was sequenced, so the mutations underlying those discordances, and the contribution of pDST limitations, remain unconfirmed. The classification of individual codon changes as disputed reflects the interpretation recorded by the testing laboratory and should be read alongside the current WHO mutation catalogue. Seventh, LPA was performed systematically only for RIF Resistance Detected and Indeterminate records and otherwise only for selected referrals, so LPA performance cannot be assessed across the full cohort. Eighth, treatment decisions and patient outcomes were not available, so we could not assess whether the proposed algorithm, or the management of patients with discordant resistance results, would improve outcomes or reduce unnecessary treatment. Finally, resistance to agents other than rifampicin was not comprehensively analysed, and MDR, pre-XDR and XDR-TB classifications should not be inferred from Ultra results alone [18].

## 5. Conclusions

In a complete annual dataset from Taiwan’s national TB reference laboratory, which serves a high-risk referral population, Xpert MTB/RIF Ultra detected MTBC with high sensitivity and had a high negative predictive value for rifampicin resistance among records with paired pDST. Culture-referenced specificity was lower among patients referred with a current or previous treatment episode, and culture-referenced positive predictive value declined from HIGH to Trace semiquantitative categories, a pattern compatible with detection of residual DNA, although differences between the specimens tested may also contribute. Rifampicin indeterminate calls were an expected consequence of Trace detection. Most pDST-discordant rifampicin resistance calls were supported by line-probe assay, but none was sequenced, so whether they reflect disputed *rpoB* mutations missed by phenotypic testing remains to be confirmed. Because most indeterminate records did not yield an interpretable reflex line-probe assay result in this workflow, stewardship strategies for Trace-derived indeterminate results should rely on clinical assessment, culture and, when indicated, a fresh specimen rather than on routine reflex LPA testing of the same specimen. The proposed algorithm requires prospective evaluation.

## Figures and Tables

**Figure 1:**
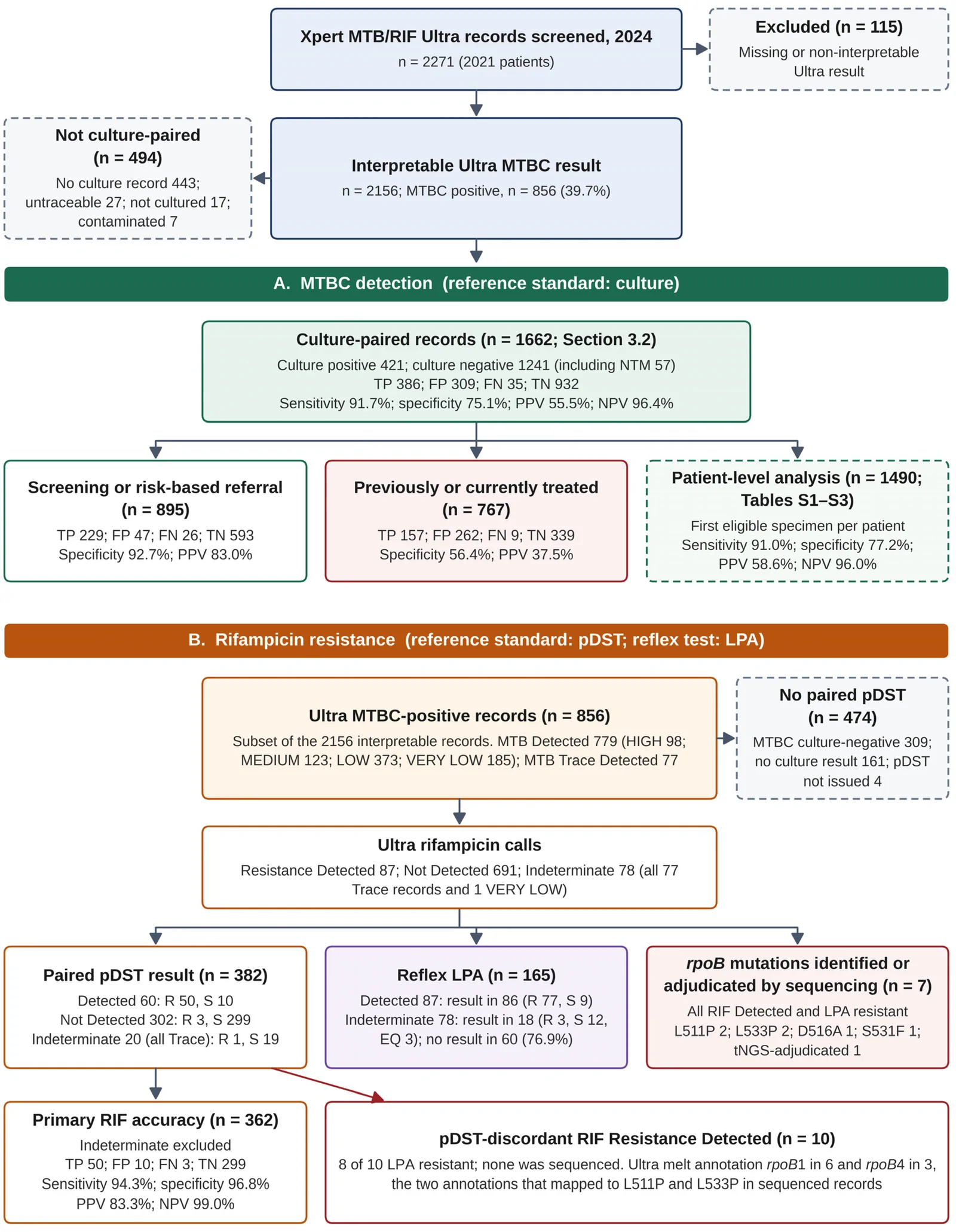
Flow diagram of specimen inclusion and analytic populations, 2024. Of 2271 screened records from 2021 patients, 2156 had an interpretable Ultra MTBC result. Panel (**A**) comprises 1662 culture-paired records, stratified by treatment history, with a patient-level sensitivity analysis. Panel (**B**) comprises 856 MTBC-positive records, of which 382 had a paired pDST result, 165 were routed for reflex line-probe testing, and 7 carried an *rpoB* mutation identified or adjudicated by sequencing. Dashed boxes denote records not entering an analysis or a sensitivity analysis. Box colors group related analyses: green, MTBC detection (Panel (**A**)); orange, rifampicin resistance (Panel (**B**)); red, the previously or currently treated stratum and the rifampicin-discordant or sequenced records; purple, reflex line-probe testing. EQ, equivocal; FN, false negative; FP, false positive; LPA, line-probe assay; MTBC, *Mycobacterium tuberculosis* complex; NPV, negative predictive value; NTM, non-tuberculous mycobacteria; pDST, phenotypic drug-susceptibility testing; PPV, positive predictive value; R, resistant; RIF, rifampicin; S, susceptible; tNGS, targeted next-generation sequencing; TN, true negative; TP, true positive.

**Figure 2:**
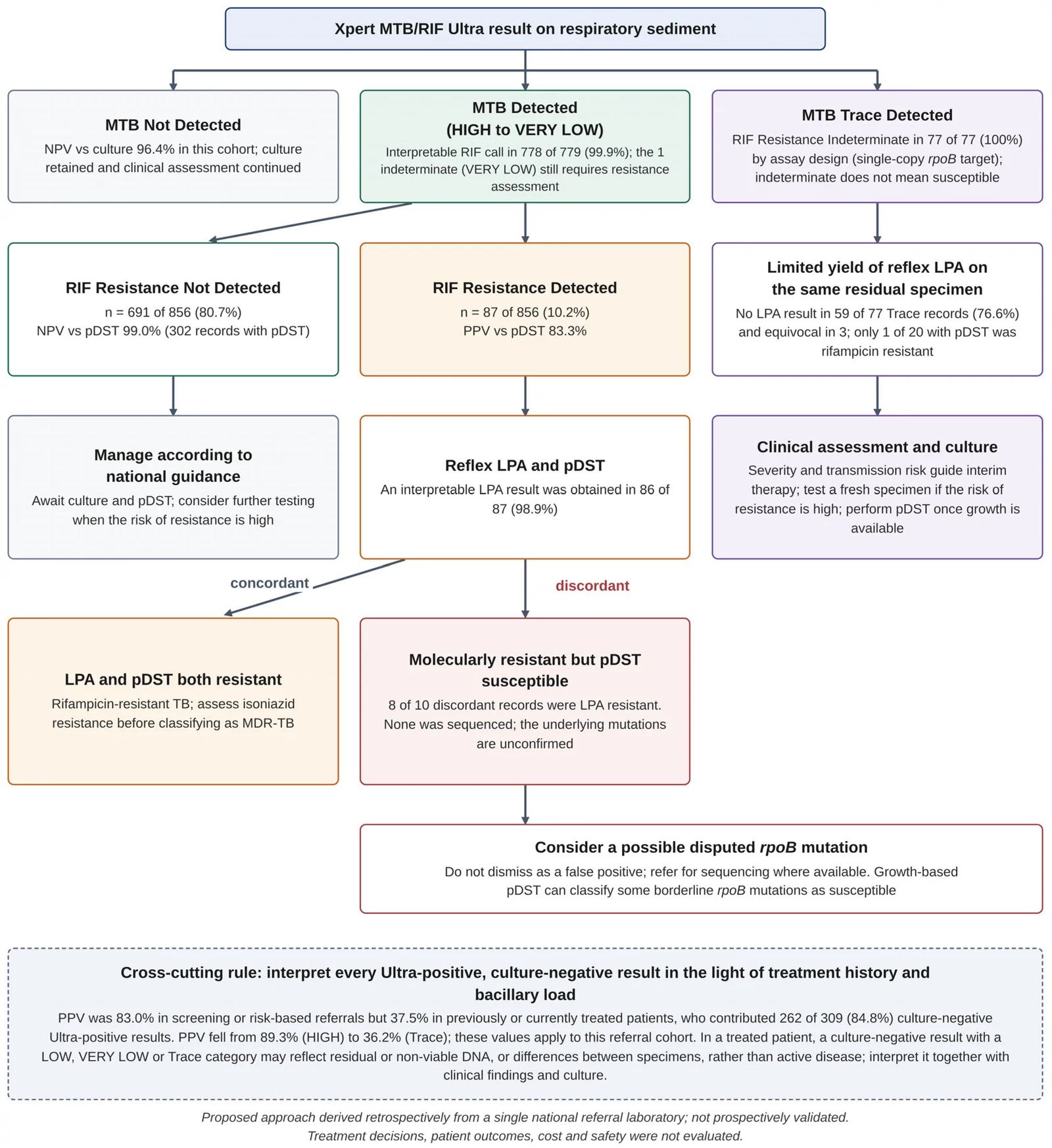
Proposed diagnostic-stewardship algorithm for Xpert MTB/RIF Ultra results in a high-risk referral setting. The algorithm was derived retrospectively from this cohort and has not been prospectively validated; treatment decisions and patient outcomes were not evaluated. An indeterminate or unsuccessful confirmatory result does not exclude rifampicin resistance. Branch points incorporate the Ultra MTBC and rifampicin results, the semiquantitative bacillary load and the observed feasibility of reflex line-probe testing; treatment history is applied as a cross-cutting rule. Box colors indicate the pathway: grey, MTB Not Detected and routine management; green, MTB Detected; orange, RIF Resistance Detected; red, pDST-discordant results; purple, MTB Trace Detected. The dashed box denotes the cross-cutting rule. Numerical annotations are drawn from the present cohort and apply to records with the relevant reference result. LPA, line-probe assay; MDR, multidrug-resistant; MTB, *Mycobacterium tuberculosis*; NPV, negative predictive value; pDST, phenotypic drug-susceptibility testing; PPV, positive predictive value; RIF, rifampicin; TB, tuberculosis.

**Table 1:** Referral categories among Xpert MTB/RIF Ultra specimen records, 2024 (*n* = 2271).

Referral Category	Records, *n* (%)	Treatment-History Group
Origin from or residence in a high MDR-TB-burden country	587 (25.8)	Screening or risk-based
Residence in a domestic high-incidence district	555 (24.4)	Screening or risk-based
Relapse or re-treatment	266 (11.7)	Previously or currently treated
Culture positivity ≥2 months after treatment initiation	254 (11.2)	Previously or currently treated
Treatment failure	179 (7.9)	Previously or currently treated
Application for second-line anti-TB drugs	158 (7.0)	Previously or currently treated
RIF resistance detected before referral	85 (3.7)	Previously or currently treated
Latent TB infection progressing to active disease	79 (3.5)	Screening or risk-based
Treatment default or loss to follow-up	39 (1.7)	Previously or currently treated
Livestock or abattoir workers with zoonotic exposure	19 (0.8)	Screening or risk-based
Close contact with an MDR-TB patient	18 (0.8)	Screening or risk-based
Recommendation by a clinical TB review committee	16 (0.7)	Screening or risk-based
Suspected or confirmed *Mycobacterium bovis* infection	16 (0.7)	Screening or risk-based
Total	2271 (100.0)	

MDR, multidrug-resistant; RIF, rifampicin; TB, tuberculosis. Records were classified according to the primary referral category documented at the time of testing; categories were treated as mutually exclusive.

**Table 2:** Sensitivity of Xpert MTB/RIF Ultra for MTBC detection by smear grade, culture-paired population (*n* = 1662).

Smear Grade	Records, *n*	Culture-Positive, *n*	Ultra-Positive Among Culture-Positive, *n*	Sensitivity, % (95% CI)
Negative	1058	104	76	73.1 (63.8–80.7)
Scanty	91	34	31	91.2 (77.0–97.0)
1+	229	91	89	97.8 (92.3–99.4)
2+	105	63	62	98.4 (91.5–99.7)
3+	67	46	46	100.0 (92.3–100.0)
4+	76	65	64	98.5 (91.8–99.7)
Not recorded	36	18	18	100.0 (82.4–100.0)

CI, confidence interval; MTBC, *Mycobacterium tuberculosis* complex. Denominators for each stratum are culture-positive records within that stratum. Smear grade was not documented in 36 records.

**Table 3:** MTBC detection accuracy of Xpert MTB/RIF Ultra stratified by treatment history.

Parameter	Screening or Risk-Based Referral (*n* = 895)	Previously or Currently Treated (*n* = 767)
True positive, *n*	229	157
False positive, *n*	47	262
False negative, *n*	26	9
True negative, *n*	593	339
Sensitivity, % (95% CI)	89.8 (85.5–92.9)	94.6 (90.0–97.1)
Specificity, % (95% CI)	92.7 (90.4–94.4)	56.4 (52.4–60.3)
PPV, % (95% CI)	83.0 (78.1–86.9)	37.5 (33.0–42.2)
NPV, % (95% CI)	95.8 (93.9–97.1)	97.4 (95.2–98.6)
Accuracy, % (95% CI)	91.8 (89.9–93.5)	64.7 (61.2–68.0)

CI, confidence interval; NPV, negative predictive value; PPV, positive predictive value.

**Table 4:** Positive predictive value of Xpert MTB/RIF Ultra against culture by semiquantitative category, culture-paired population.

Ultra Semiquantitative Category	Culture-Positive, *n*	Culture-Negative, *n*	Total, *n*	PPV, % (95% CI)
HIGH	75	9	84	89.3 (80.9–94.3)
MEDIUM	77	28	105	73.3 (64.2–80.9)
LOW	153	148	301	50.8 (45.2–56.4)
VERY LOW	60	87	147	40.8 (33.2–48.9)
Trace	21	37	58	36.2 (25.1–49.1)
Total	386	309	695	55.5 (51.8–59.2)

CI, confidence interval; PPV, positive predictive value.

**Table 5:** Xpert MTB/RIF Ultra rifampicin calls by semiquantitative category among all MTBC-positive records (*n* = 856).

Ultra Semiquantitative Category	RIF Resistance Detected, *n*	RIF Resistance Indeterminate, *n*	RIF Resistance Not Detected, *n*	Total, *n*
HIGH	17	0	81	98
MEDIUM	11	0	112	123
LOW	31	0	342	373
VERY LOW	28	1	156	185
Trace	0	77	0	77
Total	87	78	691	856

RIF, rifampicin. Trace results are not assigned a semiquantitative category and are reported as RIF Resistance Indeterminate by assay design.

**Table 6:** Characteristics of the 10 Xpert MTB/RIF Ultra RIF Resistance Detected results classified as susceptible by phenotypic DST.

Record	Ultra Load	Smear Grade	Ultra *rpoB* Melting Annotation	LPA RIF Result	LPA *rpoB* Band Pattern
1	HIGH	4+	*rpoB*4 Mut melt B	Resistant	WT8(−)
2	HIGH	3+	*rpoB*4 Mut melt B	Resistant	WT8(−)
3	HIGH	4+	*rpoB*1 Mut melt	Resistant	WT1(−)
4	MEDIUM	3+	*rpoB*4 Mut melt B	Resistant	WT8(−)
5	LOW	Negative	*rpoB*1 Mut melt	Resistant	MUT2A(+)
6	LOW	Scanty	*rpoB*1 Mut melt	Susceptible	No band loss
7	LOW	Scanty	*rpoB*1 Mut melt	Susceptible	No band loss
8	VERY LOW	1+	*rpoB*1 Mut melt	Resistant	WT1 weak positive
9	VERY LOW	Negative	Not recorded	Resistant	WT3(−)
10	VERY LOW	Negative	*rpoB*1 Mut melt	Resistant	WT1(−)

DST, drug-susceptibility testing; LPA, line-probe assay; RIF, rifampicin; Ultra load, Ultra semiquantitative category. Records are numbered serially across Table 6 and Table 7. Records 1, 2, 4 and 5 also showed *katG* WT(−)/MUT1(+), indicating concurrent isoniazid resistance. None of these records was sequenced. Ultra *rpoB* annotations are reproduced as reported by the instrument: *rpoB*1 to *rpoB*4 denote the four *rpoB* probes, and “Mut melt” denotes a melting temperature shifted from the wild-type value (“B” is the instrument’s melt-type designation). LPA band notation: WT, wild-type probe; MUT, mutation probe; (−), band absent; weak positive, band present at reduced intensity; (+), band present; no band loss, all wild-type bands present without a mutation band. MUT2A corresponds to H526Y (*E. coli* numbering).

**Table 7:** Records with an *rpoB* mutation identified or adjudicated by sequencing outside the routine workflow (*n* = 7).

Record	Ultra Load	Smear Grade	Ultra *rpoB* Melting Annotation	LPA *rpoB* Band Pattern	Documented Codon Change	Phenotypic RIF Result	*katG* Marker
11	HIGH	4+	*rpoB*4 Mut melt B	WT8 weak positive	L533P [L452P] (CTG>CCG)	Resistant	MUT1(+)
12	HIGH	4+	*rpoB*4 Mut melt B	WT8 weak positive	L533P [L452P] (CTG>CCG)	Resistant	MUT1(+)
13	HIGH	3+	*rpoB*3 Mut melt	WT8(−)	S531F [S450F] (TCG>TTT)	Resistant	Wild type
14	VERY LOW	1+	*rpoB*1 Mut melt	WT2 weak positive	L511P [L430P] (CTG>CCG)	Not available (culture negative)	MUT1(+)
15	HIGH	Not recorded	*rpoB*1 Mut melt	WT2 weak positive	L511P [L430P] (CTG>CCG)	Not available (culture negative)	Wild type
16	VERY LOW	Negative	*rpoB*2 Mut melt	WT3,4 weak positive	D516A [D435A] (GAC>GCC)	Not available (culture negative)	Wild type
17	MEDIUM	1+	Not recorded	WT2,3(−)	Not specified; adjudicated as disputed by tNGS	Superseded by tNGS adjudication	MUT1(+)

LPA, line-probe assay; RIF, rifampicin; tNGS, targeted next-generation sequencing; Ultra load, Ultra semiquantitative category. Annotation and band notation as in Table 6. Codons are given in *Escherichia coli* numbering, which is used by the assay manufacturers, with *M. tuberculosis* numbering in brackets. All seven records were reported as RIF Resistance Detected by Ultra and as rifampicin-resistant by LPA, and none is among the 10 records in Table 6. Records 11 to 16 carried a documented codon change; record 17 had a tNGS-adjudicated disputed classification without a specified codon and was excluded from the paired pDST analysis because no conventional phenotypic result was issued. The testing laboratory classified all seven as disputed; of the documented changes, only L511P and L533P correspond to the WHO borderline mutations L430P and L452P, whereas D516A and S531F are not listed as borderline.

**Table 8:** Availability and results of line-probe assay testing by Ultra rifampicin call among MTBC-positive records (*n* = 856).

Ultra RIF Call	LPA Resistant, *n*	LPA Susceptible, *n*	LPA Equivocal, *n*	No LPA Result, *n*	Total, *n*
RIF Resistance Detected	77	9	0	1	87
RIF Resistance Indeterminate	3	12	3	60	78
RIF Resistance Not Detected	0	54	0	637	691
Total	80	75	3	698	856

LPA, line-probe assay; RIF, rifampicin. RIF Resistance Not Detected records were not routed for reflex testing; LPA results in that row reflect testing performed outside the reflex rule, mainly for second-line drug applications.

## Data Availability

The data presented in this study are available on request from the corresponding author. The data are not publicly available owing to patient-privacy protection.

## Supplementary Materials

The following supporting information can be downloaded at: https://www.mdpi.com/article/10.3390/diagnostics16213651/s1, Table S1: MTBC detection accuracy of Xpert MTB/RIF Ultra against culture at the record level and at the patient level; Table S2: Patient-level MTBC detection accuracy stratified by treatment history (*n* = 1490); Table S3: Patient-level rifampicin calls by Xpert MTB/RIF Ultra compared with phenotypic drug-susceptibility testing (*n* = 355).
