# Supplementary material for: Interpreting Xpert MTB/RIF Ultra in a High-Risk Tuberculosis Referral Cohort in Taiwan: Treatment-Associated Culture Discordance and Rifampicin-Indeterminate Results
Source: Diagnostics (Basel). 2026 Nov 4;16(21):3651. doi: 10.3390/diagnostics16213651 (PMC13762257; doi:10.3390/diagnostics16213651)
Supplement: Supplementary file 1 [file diagnostics-16-03651-s001.zip › diagnostics-4617637-supplementary.pdf]

Supplementary Materials

Interpreting Xpert MTB/RIF Ultra in a High-Risk Tuberculosis Referral Cohort in Taiwan: Treatment-Associated Culture Discordance and Rifampicin-Indeterminate Results

Ming-Jr Jian [1,2,†], Hsing-Yi Chung [1,†], Tai-Han Lin [1], Chih-Kai Chang [1], Cherng-Lih Perng [1,*] and Hung-Sheng Shang [1,*]

Methods for the patient-level sensitivity analysis

The main analyses were performed at the specimen-record level and retained duplicate submissions. To assess whether repeated testing of the same patient influenced the estimates, each analysis was repeated after restricting the dataset to the first eligible specimen per patient, identified by an innominate patient key and ordered by date of receipt and laboratory accession number. The 1662 culture-paired records originated from 1490 unique patients, and the 382 records with a paired phenotypic rifampicin result originated from 355 unique patients. Treatment-history groups were defined as in the main text. Confidence intervals are Wilson 95% intervals.

Table S1. MTBC detection accuracy of Xpert MTB/RIF Ultra against culture at the record level and at the patient level.

Analysis level	n	TP	FP	FN	TN	Sensitivity, % (95% CI)	Specificity, % (95% CI)	PPV, % (95% CI)	NPV, % (95% CI)
Record level (main analysis)	1662	386	309	35	932	91.7 (88.7–94.0)	75.1 (72.6–77.4)	55.5 (51.8–59.2)	96.4 (95.0–97.4)
Patient level, first eligible specimen	1490	355	251	35	849	91.0 (87.8–93.5)	77.2 (74.6–79.6)	58.6 (54.6–62.4)	96.0 (94.5–97.1)

CI, confidence interval; FN, false negative; FP, false positive; NPV, negative predictive value; PPV, positive predictive value; TN, true negative; TP, true positive.

Table S2. Patient-level MTBC detection accuracy stratified by treatment history (n = 1490).

Treatment-History Group	n	TP	FP	FN	TN	Sensitivity, % (95% CI)	Specificity, % (95% CI)	PPV, % (95% CI)	NPV, % (95% CI)
Screening or risk-based referral	830	221	42	26	541	89.5 (85.0–92.7)	92.8 (90.4–94.6)	84.0 (79.1–88.0)	95.4 (93.4–96.9)
Previously or currently treated	660	134	209	9	308	93.7 (88.5–96.7)	59.6 (55.3–63.7)	39.1 (34.1–44.3)	97.2 (94.7–98.5)

Abbreviations as in Table S1. The corresponding record-level estimates are given in Table 3 of the main text.

Table S3. Patient-level rifampicin calls by Xpert MTB/RIF Ultra compared with phenotypic drug-susceptibility testing (n = 355).

Ultra RIF Call	pDST Resistant, n	pDST Susceptible, n	Total, n
RIF Resistance Detected	41	8	49
RIF Resistance Indeterminate	1	19	20
RIF Resistance Not Detected	2	284	286
**Total**	**44**	**311**	**355**

After exclusion of the 20 indeterminate results (n = 335): sensitivity 95.3% (95% CI 84.5–98.7), specificity 97.3% (95% CI 94.7–98.6), PPV 83.7% (95% CI 71.0–91.5), NPV 99.3% (95% CI 97.5–99.8).

NPV, negative predictive value; pDST, phenotypic drug-susceptibility testing; PPV, positive predictive value; RIF, rifampicin. The corresponding record-level estimates are given in Section 3.6 and Figure 1 of the main text.
